# Impact of Fava Bean (*Vicia faba*) Processing on Quality Characteristics and Digestibility of a Protein-Rich Snack

**DOI:** 10.3390/foods13152372

**Published:** 2024-07-26

**Authors:** Kateryna Khvostenko, Sara Muñoz-Pina, Jorge García-Hernández, Ana Heredia, Ana Andrés

**Affiliations:** 1 Instituto Universitario de Ingeniería de Alimentos para el Desarrollo (FoodUPV), Universitat Politècnica de València, Camino de Vera s/n, 46022 Valencia, Spainaandres@tal.upv.es (A.A.); 2 Centro Avanzado de Microbiología de Alimentos (CAMA), Universitat Politècnica de València, Camino de Vera s/n, 46022 Valencia, Spain

**Keywords:** fava beans, snack, fermentation, *Pleurotus ostreatus*, protein, digestibility

## Abstract

The impact of fava bean processing methods (soaking, autoclaving, fermentation) on a legume-based bars’ quality, protein characteristics, and digestibility was shown. The antioxidant and the angiotensin-converting enzyme-inhibitory capacity before and after in vitro digestion were investigated to reveal the potential advantages of fava bean usage for snacks. All bars have demonstrated high protein content, varying from 22.1 to 25.1 g/100 g DB. Based on the fermented fava beans of *Pleurotus ostreatus*, the samples were characterized by a higher concentration of essential amino acids by 8.6% and a reduction of tannins by 18.5% compared with bars based on soaked fava beans. Sensory evaluation improved the color, texture, and overall acceptability of the bars with fermented legumes. Various types of bean processing did not significantly affect the protein digestibility of the bars. The fermentation method positively affected the angiotensin-converting enzyme-inhibitory properties of bars and increased by 16.5% (before digestion) and 15% (after digestion) compared with other samples. After digestion, samples were characterized by a high level of Fe bioaccessibility (100, 83, and 79% for the bars based on soaked, autoclaved, and fermented fava beans, respectively) and increased total phenolic content. These findings highlight the potential health benefits of fava bean usage for snack products.

## 1. Introduction

Nowadays, the childhood obesity problem is a major concern for modern society. According to WHO reports [1], over 390 million children and adolescents aged 5–19 years were overweight in 2022, including 160 million who were living with obesity. It is known that gaining extra weight for children and adolescents is usually related to serious comorbidities that can continue into adulthood, including cardiovascular dysfunction and type 2 diabetes, as well as social and psychological issues [2,3]. A plethora of studies have revealed that the consumption of unhealthy, energy-dense food, characterized by a high content of fast carbohydrates, saturated fats, and reduced amounts of fiber and protein, are among the main factors for childhood obesity development [4]. Such foods are associated with snacks and “food-to-go” for most consumers. At the same time, based on the literature review, it is evident that the term “snack” still does not have clear and steady clarification for researchers and food producers. Different authors use this definition in a variable manner, which affects the interpretation of scientific results and the practical application of presented findings.

It is worth mentioning that Kaisari et al. [5] and Marangoni et al. [6] reported results revealing that higher eating frequencies are associated with lower weight status in children and adolescents in the case of including healthy food options in children’s diets for snacking. Such data are aligned with Juton et al. [7], which showed an inverse trend between meal frequency and the incidence of excessive weight and abdominal obesity among children in Spain. Due to this fact, balanced specific patterns of snacking could positively impact children´s health, as 92.9 to 100.0% of children consume at least one snack per day [4].

It is known that protein presence in a diet is crucial for the optimal growth of children and adolescents, and its intake plays a key role in their metabolic health status and extra weight-gaining process [8]. Recent studies have highlighted probable evidence for an association between a higher intake of animal protein and increased body mass index among the younger generation [9,10]. Based on this, plant protein-rich ingredients have gained significant attention as their usage could be a perspective strategy to substitute animal-based proteins and make shifts towards healthier and more sustainable food choices for consumers. For this purpose, Shah et al. [11] have suggested developing corn-based extruded snacks enriched with soybean and chickpea flour as plant protein sources. The developed products with soybean flour were characterized by higher protein and dietary fiber content. However, at the same time, it was mentioned that using extrusion as the processing method led to the decrease of some amino acids, such as lysine, isoleucine, etc., which are vital for children’s development [1]. Also, to improve the nutritional value of the snacks and increase the presence of plant protein, *W. globosa* (duckweed) was suggested [12]. The authors have shown that the addition of this ingredient greatly enhanced the nutritional value of the final products. Considering that such ingredients are not typical for consumers, sensory evaluation of the products should also be performed.

Among the potential of plant-based sources for the protein enrichment of snacks, fava beans have been characterized by increased interest among researchers and food producers over the last few decades. It is a leguminous cold-tolerant crop, which ranks among the most grown and consumed legumes worldwide due to its exceptional nutritional profile and ecological benefits [13]. Growing fava beans, which is characterized by wide adaptability to a range of soil environments, could be related to a sustainable approach in the agricultural economy and has a positive impact on the environment in terms of their ability to engage in symbiotic nitrogen fixation processes, which reduce the reliance on synthetic nitrogen fertilizers [14]. Fava beans contain a significant content of proteins (25–40%); dietary fiber; a notable content of resistant and slowly digestible starch; a variety of minerals such as magnesium, zinc, potassium, phosphorus, and iron; and bioactive compounds, such as total phenolics and flavonoids [15].

Considering the versatile potential of the mentioned legume, a significant increase in studies focusing on using fava beans to fortify protein content in different food commodities has also been observed. Previous studies have shown the possibility of incorporating raw bean flour into bread (8%) and gluten-free biscuit production [13]. Boukid et al. [16] reported on incorporating fava bean protein concentrate or isolate in the durum wheat pasta recipe (25%). The authors studied the usage of fava beans for extruded snack production, but the primary focus was on the extrusion effects on techno-functional properties. The authors pointed out the limited consumer acceptability of products with high fava bean content due to sensory bottlenecks like taste, aroma, and color.

Besides the sensory aspects, several antinutritional factors in fava beans have hindered their usage as a superior plant protein ingredient in food production [17]. Different technological techniques have been suggested to reduce antinutrients in fava bean content and to increase its utilization in the food industry. Among them are non-thermal (dehulling and soaking), thermal (cooking, autoclaving, microwaving, and extrusion), and biological (germination and fermentation) [18]. The results have revealed the effectiveness of various processing methods in improving the nutritional potential of fava beans for use in diverse protein-rich products. At the same time, it should be mentioned that despite the benefits of the fava bean processing methods, the effect of any treatments on the quality and nutritional potential of the final products enriched with this plant protein source has been scarcely investigated. Therefore, research aiming to mitigate and eliminate undesirable characteristics and enhance the overall nutritional and functional quality of fava bean-based products is still in high demand.

In this context, this study aimed to evaluate the effect of processing methods (soaking, autoclaving, and fermentation) on the quality and functionality characteristics (such as texture and sensory evaluation, protein characteristics, antioxidant activity, and ACE inhibitory activity) and digestibility of fava bean-based snacks (bars).

## 2. Materials and Methods

### 2.1. Materials

Fava beans (*Vicia faba* L.) from Batlle^®^ were obtained from local stores in Valencia (Spain). Puffed quinoa, pumpkin seeds, carob syrup, and konjac gum were purchased at a local supermarket in Valencia (Spain). The *Pleurotus ostreatus* strain was acquired from the Spanish Type Culture Collection (CECT20311).

For the different determinations, the following reagents were used: sulfuric acid, sodium hydroxide, sodium chloride, thioglycolic acid, phytic acid, 2,2′-bipyridine, 4-dimethylamino benzaldehyde, acetylacetone, ascorbic acid, ethyl acetate, acid formic, 2,2-diphenyl-1-picrylhydrazyl (DPPH), 2,2′-azino-bis (3-ethylbenziothiazoline-6-sulfonic acid) (ABTS), Folin–Ciocalteu reagent, 2,4,6-tripyridyl-s -triazine (TPTZ), gallic acid, (±)-6-hydroxy-2,5,7,8-tetramethylchroman-2-carboxylic acid (Trolox), N-Hippuric-His-Leu glucose hydrate, mycopeptone, and chloramphenicol. These were all obtained from Sigma-Aldrich Co. (Saint Louis, MO, USA). For digestion assays, pepsin from porcine gastric mucosa (3200–4500 U/mg, P6887), pancreatin (8 × USP, P7545) from porcine pancreas, p-toluene-sulfonyl-L-arginine methyl ester (TAME, T4626), bovine bile (dry, unfractionated, B3883), analytical grade salts, hydrochloric acid (37%), sulfuric acid (95–97%), sodium hydroxide, D-+-glucose (≥99.5%), ethanol (96%), and baker’s yeast invertase (Grade VII, ≥300 units/mg solid, I4504) were also obtained from Sigma-Aldrich Co. In addition, glacial acetic acid, concentrated hydrochloric acid, absolute ethanol, ethyl ether, sodium carbonate, ammonium iron (III) sulfate dodecahydrate, and EDTA calcium disodium salt were obtained from Panreac AppliChem (Barcelona, Spain). Nitric acid (70%), acetonitrile (HPLC grade), methanol (HPLC grade), iron (III) chloride hexahydrate, sodium acetate trihydrate, and potassium persulfate were obtained from Honeywell Fluka (Morris Plains, NJ, USA).

### 2.2. Methods

#### 2.2.1. Fungal Solid-State Fermentation (SSF) of Fava Beans

Before inoculation, beans were soaked for 16 h at 22 ± 2 °C in a 1:6 ratio to achieve a final moisture of 55%. Afterward, beans were crushed using a food processor (Thermomix^®^, TM6-1, Wuppertal, Germany), applying 5000 rpm for 10 s and 10 s more at 10,000 rpm, reaching a particle size of 0.5 ± 0.1 cm. Then, they were blanched for 2 min and cooled with water. Subsequently, fava beans were autoclaved (Vertical Support Autoclave 4002136, JP Selecta™, Barcelona, Spain) for 20 min at 121 °C. It should be noted that the *Pleurotus ostreatus* starter preparation was performed using the method described by Muñoz-Pina et al. [19]. Three different samples were obtained to study the effect of the various processing steps on the protein-rich bars’ characteristics: soaked fava beans (soaked beans); soaked, autoclaved, and incubated fava beans without inoculum (autoclaved beans); and soaked, autoclaved, and fermented fava beans (fermented beans). The nomenclature refers to the last step to which the samples are subjected. At least three independent replicates were studied for each case.

#### 2.2.2. Selection of Snack Type According to Consumer’s

A fava bean-based bar was selected to be developed because it fits the main requirements, needs, and facilitators for acceptance according to a previous study with parents and children (6–12 years old). Focus group sessions with parents revealed that healthy snacks for children should be satiating, rich in fiber, easy to carry, contain natural ingredients, and not stain children’s fingers. Co-creation sessions with children (6–12 years old), including different creative activities, such as emphatic design, analogies, and brainstorming, were used to generate new prototypes of products. The creative designs of legume snacks generated by children included bar-like products and sticks that, according to children, would be better accepted if they had funny shapes (smiley faces, soccer balls, cups), formats (e.g., the package could contain several units for sharing with friends), and flavor (chocolate, spicy).

#### 2.2.3. Fava Bean-Based Bar Formulation

The Design-Expert (v.12) software was used to determine the optimum proportions of fava bean-based bar formulation. The mixture design (D-optimal) was used to optimize the mixture levels of three main ingredients: fava beans (50–57%), puffed quinoa (15–20%), and pumpkin seeds (11–18%). The effects of the main ingredient’s ratio on the sensory attributes of bars were investigated, and the optimum mixture was selected as follows: fava beans—50%; puffed quinoa—16%; and pumpkin seeds—14%. Also, the bars’ recipe includes carob syrup (20%) and konjac gum (1%).

Three types of protein-rich snacks were designed: bars with soaked fava beans (SFB), bars with autoclaved fava beans (AFB), and bars with fermented fava beans by *Pleurotus ostreatus* (FFB).

Bars were prepared as follows: The konjac gum was diluted in water with a 1:15 ratio; then, all ingredients were mixed for 4 min. The prepared dough mass was rolled to the thickness of 3 cm, and the mass was shaped in bars, cutting into pieces of 10 cm × 3.3 cm × 3.1 cm. Afterward, bars were baked in an oven with air circulation (at 125 ± 2 °C) for 35 min, then covered and left to cool at room temperature for 1 h. Physical analyses (color and hardness) were performed after 24 h of storage. Some of the bar samples were frozen to be used for chemical composition, antioxidant capacity, and in vitro digestibility analyses.

#### 2.2.4. Static In Vitro Gastrointestinal Digestion

Fava bean-based bars underwent in vitro digestion using the standardized INFOGEST method, as defined by Brodkorb et al. [20], ensuring all enzyme activity was as described in the study’s supplemental information. Briefly, 5 g of each bar was mixed with 5 mL of simulated salivary fluid, including α-amylase (75 U/mL), at pH = 7 for 2 min at 37 °C (chamber JP Selecta SA, Barcelona, Spain) under gentle stirring at 25 rpm using an Intelli-Mixer RM-2 (Elmi Ltd., Riga, LV-1006, Riga, Latvia). Subsequently, 10 mL of simulated gastric fluid with pepsin enzyme (2000 U/mL) was added, and the pH was adjusted to 3 using HCL. The mixture was kept under stirring at 55 rpm for 2 h at 37 °C. In the final intestinal digestion stage, 20 mL of simulated intestinal fluid containing pancreatin (with 100 U/mL trypsin activity) and bile salts (10 mM) was incorporated, and the pH to 7.0 was adjusted with NaOH. The mixture was left at 37 °C for 2 h with continuous stirring at 55 rpm. Bowman–Birk Inhibitor (BBI) at 0.05 g/L was added to each tube to stop the enzyme activity. Afterward, the bioaccessible fraction (supernatant) was taken after centrifugation at 8000× *g* for 10 min at 4 °C and kept at −40 °C for further analysis. A control with only the fluids and enzymes was also studied.

#### 2.2.5. Analytical Determinations

##### Optical Properties

A spectro-colorimeter (Minolta, CM-3600D, Minolta Co LTD, Japan) was used to measure the color of the different bars, considering a standard illuminant D65 and a standard observer of 10◦. CIE-L*a*b* color coordinates were measured, and tone (h) and chroma (C*) values were calculated by the device with the a* and b* coordinates. Color differences (∆E) were calculated according to the following equation (Equation (1)):

(1) $$∆E=\sqrt{{\left({∆L}^{*}\right)}^{2}+{\left({∆a}^{*}\right)}^{2}+{\left({∆b}^{*}\right)}^{2}}$$

##### Mechanical Properties

The textural quality of bar samples was measured with a cut test (CTT) performed using a TA-HD texture analyzer (Stable Micro Systems XT-RA). The hardness value (g) was the peak force that occurred during the first compression. Three cereal bars per formulation were tested at 2 cm from each edge lengthwise. The operating conditions for the hardness measurements were as follows: 1.0 mm/s pretest speed and 0.5 mm/s test speed.

##### Compositional Analysis

Moisture, protein, lipid, ash, and carbohydrate contents were analyzed according to the Association of Official Analysis Chemists International [21], the last one by difference as total carbohydrates = 100 − (proteins + lipids + ash). The results were expressed in g per 100 g of dry matter. Furthermore, the DNS colorimetric method was used to determine the initial reducing sugar content [22]. The energy value was estimated considering 4 kcals per gram of carbohydrates and proteins and 9 kcals per gram of lipids.

##### Mineral Quantification

The concentrations of three minerals—iron (Fe), calcium (Ca), and magnesium (Mg)—in fava bean bars were measured both before and after gastrointestinal (GI) digestion using Inductively Coupled Plasma Mass Spectrometry (ICPMS7900, Agilent). The preparation of the mineral extract followed the protocol outlined by Sánchez-García et al. [23]. For this, 5 g of bars or 3.5 mL of the bioaccessible fraction from digested samples were heated at 550 °C until white ashes were obtained. Afterward, the ashes were dissolved in 1.5 mL of 69% nitric acid and diluted with 4 mL of bi-distilled water before analysis by ICP-MS. Results were expressed in mg/100 g dry basis.

##### Determination of Total Phenolic Content (TPC), Antioxidant Activities, and Condensed Tannin Content (CTC)

The TPC content, antioxidant activity, and CTC were studied before and after GI digestion. A previous ethanolic extraction was carried out for undigested bars following the protocol described by Caprioli et al. [24], with some modifications. A sample of 5 g was mixed with 20 mL of 70% ethanol at a pH of 2. This mixture was then maintained under agitation for 120 min at 20 °C. The tube was centrifuged at 8000× *g*-force for 15 min. This extraction step was performed twice, combining the supernatants into one sample, which was then passed through a 0.45 μm polytetrafluoroethylene (PTFE) filter and kept in a freezer until further analysis. After luminal digestion, an aliquot of the digest was taken to perform the different analyses.

The TPC was quantified using the Folin–Ciocalteu method, as modified by Muñoz-Pina et al. [25]. The results were expressed in mg of gallic acid equivalent (GAE) per g dry basis (DB). Antioxidant activity was evaluated using three distinct assays, ABTS, DPPH, and FRAP, following the protocol described by Thaipong et al. [26] and expressed in mg of Trolox per g DB.

Finally, the CTC of the bars was assessed using the acidified vanillin assay [27], where tannin contents were reported as catechin equivalents (mg CAE per g DB).

##### Amino Acid Analysis by High-Performance Liquid Chromatography

The procedure used for the amino acid extraction before free amino acid content analysis in the undigested bars was performed by two different hydrolysis methods: alkaline and acid hydrolysis [28]. To measure all amino acids except tryptophan, a mixture of 10 mL of 6 M HCL and 0.2 g of the sample was heated at 110 °C for 24 h. Once cooled to room temperature, the mixture was passed through a 0.2 μm cellulose acetate filter. A 200 μL portion of the resulting acid extract was completely dried in a vacuum oven (JP Selecta, 0493583, Barcelona, Spain) at 100 °C. The dry sample was subsequently rehydrated with 40 μL of 20 mM HCl, preparing it for the derivation process. Alkaline extraction was carried out to measure tryptophan. Briefly, 0.2 g of the sample was combined with 10 mL of 4.3 M LiOH·H_2_O, sealed in a tube under nitrogen, and heated at 120 °C for 16 h. After cooling, this mixture was also filtered using a 0.2 μm cellulose acetate filter before derivatization.

In the case of the bioaccessible fraction of the digested samples, no previous extraction was carried out, as it was required that free amino acids were measured. Thus, a 1 mL aliquot from digested samples underwent filtration through a 0.2 μm cellulose acetate filter before derivatization.

Both derivatizations of extracts/digests and analysis in the HPLC were conducted with the Waters AccQ-Tag kit. The analysis of amino acids was carried out using a 1200 Series Rapid Resolution HPLC connected to a Series diode array detector (Agilent, Palo Alto, CA, USA). Chromatographic separation of amino acids was achieved on an AccQ-Tag column (3.9 × 150 mm) maintained at 37 °C, with a flow rate of 1 mL/min and an injection volume of 5 μL. The mobile phase consisted of Eluent A (90:10, bi-distilled water: AccQ-Tag Eluent A) and Eluent B (60:40, ACN: bi-distilled water). Unknown compounds were identified by comparing their retention times to reference standards at a wavelength of 250 nm. The results were expressed as mg of amino acid per g of dry matter.

##### Fourier Transform Infrared (FTIR) Analysis

Protein isolates of the fava bean bars were analyzed using attenuated total reflectance (ATR) Fourier transform infrared (FTIR) spectroscopy (Cary 630 spectrometer, Agilent Technologies Inc., Santa Clara, CA, USA) according to Shrestha et al. [29]. Dried protein samples were placed on the diamond cell and scanned 120 times in a range from 4000 to 650 cm^−1^ at a resolution of 4 cm^−1^ against the empty cell. This analysis was conducted in triplicate. An alkaline-assisted process was used to prepare crude protein extracts, as described by Akıllıoğlu et al. [30].

For detailed secondary structural analysis of the isolates, the band between 1600 and 1700 cm^−1^ corresponding to the Amide I band underwent Fourier self-deconvolution, second derivative analysis and curve fitting to identify overlapping peak structures using OriginPro 2024 10.1.0.170 (Learning edition) software. The analysis identified six distinct Gaussian peaks at 1638, 1654, 1663, 1680, 1617, and 1695 cm^−1^. These peaks align with β-sheet, α-helix, β-turn, anti-parallel β-sheet (β-A), and conformations indicative of protein aggregates and amino acid sidechain structures (designated A1 and A2), respectively, in concordance with other reports [31].

##### In Vitro Protein Digestibility

The extent of protein hydrolysis after the intestinal stage of digestion was evaluated by measuring the soluble protein fraction in trichloroacetic acid (TCA), as detailed in [32]. Briefly, 500 μL of 36% TCA was combined with 1 mL of the bioaccessible fraction and left for 15 min at 4 °C using an Eppendorf Thermomixer Comfort. After centrifugation at 1200× *g* for 10 min, the resulting supernatant was diluted in a 50 mM EDTA and 8M UREA buffer (pH 10), and its absorbance was measured at 280 nm. For undigested samples, 100 mg of the sample was in 0.9 mL of distilled water, followed by the addition of 0.5 mL of 36% TCA. The data were expressed as g/100 g protein after obtaining the corresponding calibration curve with L-tyrosine as a standard.

##### Angiotensin-Converting Enzyme Inhibitory Activity (ACE ia (%))

The ability of the samples to inhibit the angiotensin-converting enzyme (ACE) both before and after in vitro digestion was assessed following the procedure described by Akıllıoğlu et al. [30]. The protein isolates (same as for FTIR analysis) from undigested samples and the bioaccessible fractions from the final step of luminal digestion were evaluated for their inhibitory effects. In a typical assay, each sample was mixed with an ACE solution. Subsequently, the Hip-His-Leu substrate was introduced, and the hippuric acid reaction proceeded. The final amount of hippuric acid was quantified by measuring its absorbance at 228 nm. A control without a sample was also conducted to quantify the inhibitory capacity of samples.

#### 2.2.6. Preliminary Sensory Evaluation

The preliminary sensory analysis was conducted in the specialized laboratory for sensorial evaluation at the Instituto Universitario de Ingeniería de Alimentos para el Desarrollo (FoodUPV), Universitat Politècnica de València. The developed snacks were offered to the 25 participants for an organoleptic test using a 9-point hedonic scale (1—lowest intensity, 9—highest intensity) on appearance, color, aroma, texture, stickiness, taste, and overall acceptance, as described in [31]. Before the assessment, all participants, selected by invitation, signed a written free-consent form. To analyze sensory acceptability, all the protein-rich bars were produced on the same day and evaluated 24 h later. Samples of three different bars, formed into cuboids, were marked with randomly assigned two-digit codes and were provided on plastic plates. Between the evaluations, the participants rinsed their mouths with water.

#### 2.2.7. Statistical Analysis

All assays were performed in triplicate, and outcomes were presented as the average value with the standard deviation. Statistical evaluation of the data was carried out using Statgraphics Centurion-XV, applying one-way analysis of variance (ANOVA) with a 95% confidence level (*p* < 0.05) to determine homogeneous groups.

## 3. Results and Discussion

### 3.1. Quality and Preliminary Sensory Characterization of Developed Bars

The results of the quality characteristics of fava bean-based bars are presented in Table 1. It is necessary to say that using various processing methods for ingredients can significantly affect not only the nutritional value of the final products based on this raw material but also the quality parameters. Developing new products that resemble analogs/traditional ones in terms of color, taste, flavor, aroma, and texture will be beneficial in overcoming the challenges of legume presence limitation in a child’s diet.

The color of a food product plays a crucial role in its acceptance, especially for those based on new ingredients. This study showed that using processed fava beans for bar production affects their color parameters (Table 1). The developed bars were characterized by highly heterogeneous surfaces, as expected for such products. Including the autoclaved fava beans in the snack recipe reduces the L* value (34.81 ± 1.30) compared with the SFB (42.80 ± 1.49), respectively. The indicated darkening of the mentioned samples could be related to phenolic compounds’ oxidation, Maillard reaction, and caramelization of fava beans during autoclaving. These results agree with and confirm those obtained by Pedrosa et al. [33]. However, the color of bars with fermented fava beans is brighter (L*—37.34 ± 1.47) than AFB. A difference was also observed for the b* value. The SFB had the highest b* value (28.34 ± 1.30) compared with AFB (23.13 ± 0.34) and FFB (24.54 ± 1.022). Obtaining a darker brownish color, which can be associated with cocoa added to the samples with processed fava beans (autoclaved and fermented), could be beneficial to attract consumers [34,35].

One of the main challenges for the industry in developing legume-based food products is the significant changes in their texture, flavor, appearance, and color, which are not in accordance with consumer acceptance and preferences. Previous studies have shown that the incorporation of legumes into various products could be accompanied by the “beany” flavor of the product, which is considered “unpleasant” for panelists [36]. Salvador-Reyes et al. [13] also reported that sensory acceptability for food products with high concentrations of fava bean flour remains a notable challenge. Due to this, it must be highlighted that using legumes with various processing methods should enhance the developed products’ nutritional quality and improve their sensory properties.

**Figure 1:**
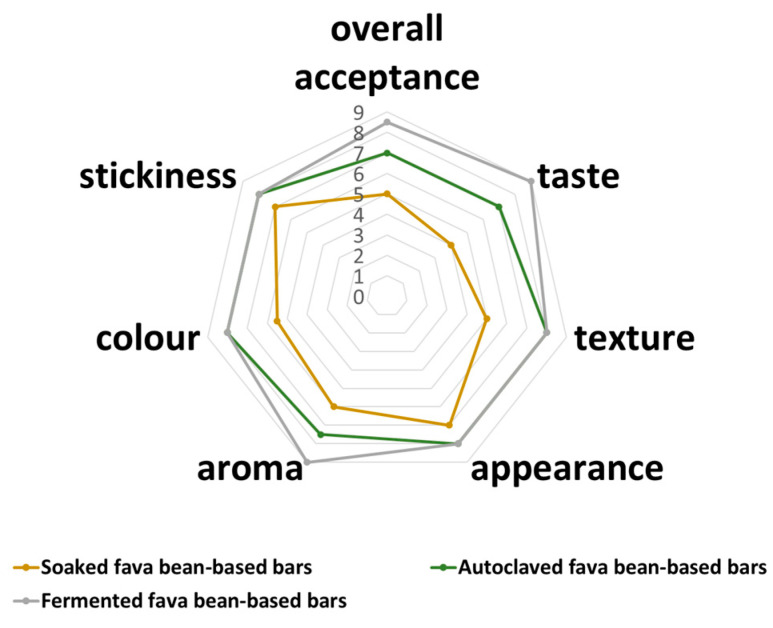
Spider web chart of bars’ hedonic evaluation.

The preliminary sensory analysis revealed that bars based on the mixture of ingredients with fermented fava beans were more appreciated than other samples (Figure 1).

The FFB sample was characterized with the highest scores for the aroma and taste characteristics compared with SFB and AFB bars. Such a tendency could result from a sequence of biochemical processes in which the starter culture produces enzymes and other metabolites that contribute to the unique flavor and aroma of the final product. Some previous studies also reported the positive effect of the LAB fermentation of several legumes (pea, lupin, fava beans) on the sensory characteristics of the final products based on them [37,38,39].

Positive changes in the AFB and FFB bar color and appearance were also observed. These samples were characterized by a more attractive brownish color that occurred because of the Maillard reaction and were in accordance with the color measurements (Table 1). The obtained tinge can be associated with cocoa or chocolate in the recipe and beneficially affect the samples’ overall acceptability for the targeted consumer group (children and adolescents).

Hardness is the most frequently determined parameter to evaluate snack texture since it usually refers to the maximum force during the first bite to break food [40]. For the SFB and AFB, the values of this parameter were 731.0 ± 54 g and 691 ± 29.0 g, respectively. The obtained value for the bars based on fermented fava beans was the lowest, 572 ± 16 g. These results could be related to the impact of the processing methods on fava bean texture as the main ingredient. Previous studies [41] have revealed that hydrothermal processing, such as autoclaving, softens a legume’s texture due to the effect on the seed coats and cotyledons. It was also reported that fermented soybeans were characterized by reduced hardness and chewiness compared with raw samples linked to microbial proteolytic activities [42]. The heating and fermentation processes, depending on the parameters and strains used, could also affect the interaction between the protein structures [43,44] and change the rate of aggregation and gelation of protein molecules, which also has an impact on the texture characteristics of the final products. Also, it should be mentioned that texture properties can be affected by enzyme-mediated crosslinking, due to the presence of laccases from fermentation processing with *Pleurotus ostreatus* [45]. Nevertheless, in real food matrices, such as bars, it is quite challenging to characterize or even predict mixed protein gel formation based on the properties of each type of protein in the mixture and their interaction with each other.

### 3.2. Nutritional Composition and Antioxidant Capacity (DPPH, ABTS, FRAP) of Fava Bean-Based Bars

The results of the proximate chemical composition of the fava-bean-based bars in terms of moisture, crude protein, lipids, total carbohydrates, reducing sugar contents, total starch, and ash are shown in Table 2. Moisture is one of the main parameters that can indicate the stability of bars during storage. The moisture varied between 23.75 and 28.25 g/100 g, depending on the processing method of fava beans. These results were similar to the findings of Sahil Gupta et al. [46], with a value ranging between 23.31 ± 1.20 and 27.81 ± 0.09 g/100 g for protein-rich bars based on chickpeas.

The protein content of the bars was not significantly affected by the type of fava bean processing method. The average protein content of the SFB, AFB, and FFB were 23.8% ± 0.7%, 22.1% ± 1.5%, and 25.1% ± 0.3%, respectively. This result is in close agreement with the findings of Jabeen et al. [47], who developed energy-rich protein bars based on cheddar cheese, whey protein isolate, roasted chickpea flour, and rice flour in different proportions with a protein content that varied from 22.3% to 23.6%. At the same time, bars containing fava bean cotyledons developed by Ziena et al. [48] were characterized by 13.0% protein. Such results are lower than in the present study.

Due to EC Reg. 1924/2006 [49], the observed amount of protein allows for labeling all developed products as “with high protein content”, and the obtained results highlight the effectiveness of fava bean usage as a diversifying plant protein source for snack production.

All bar samples showed very similar results in terms of carbohydrate content. Bars based on the fermented fava beans were characterized by a reduced sugar increase compared with samples based on the autoclaved fava beans by 25.6%. Such results are attributed to the metabolic activity of *Pleurotus ostreatus*, which degrades carbohydrates into simple sugars for their fungus growth. Similarly, Garrido-Galand et al. [50] showed the effect of the solid-state fermentation of various fungus types on the reduced sugar content of quinoa, wheat, and oats.

Concerning minerals, the usage of fava beans with different types of processing for snack production did not affect the Fe, Ca, and Mg content. Considering these results, including the developed bars in children’s diets can be a beneficial support for their growth and development and will provide an additional source of minerals.

The effect of fava bean treatment was determined on the antioxidant benefits and the bars’ phenolic and antinutrient content (tannins) (Table 2). Antinutrient compounds (including trypsin inhibitors, tannins, etc.) are one of the main factors limiting the usage of legumes as a plant protein source in the food industry. These compounds are vital for plants to protect them against pests and viruses that can cause harm and, at the same time, consumption of food with high amounts of antinutrients, which can interfere with the absorption and utilization of essential nutrients or inhibit digestive enzymes, causing negative impact on human health [18]. The highest tannin content was determined in the bars with soaked fava beans (0.97 ± 0.01 mg CAE/g DB), and the lowest value was obtained on the sample with fermented ones (0.79 ± 0.02 mg CAE/g DB). From data obtained from Table 2, fermentation with *Pleurotus ostreatus* of fava beans had the most significant effect in reducing tannin content by 18.5%. This tendency aligns with the results obtained by Badjona et al. [17] and Muñoz-Pina et al. [19].

The results demonstrate differences between the total phenolic content due to the processing of fava beans. It was found that bars with autoclaved and fermented fava beans had a reduced phenolic content of 19.4 and 34.2%, respectively. Similar results are found in the literature on different types of treatment for fava beans [19,51]. At the same time, the higher phenolic content of the SFB could be related to the deamination of tyrosine, the precursor of phenolic acids, which is present in higher concentrations in this sample.

Interactions between compounds with different action mechanisms determine the food product’s antioxidant capacity. For this reason, the antioxidant capacity of bars was determined by different complementary methods, which evaluated various mechanisms of action—DPPH, FRAP, and ABTS assays. The results showed that snack bars’ TPC and antioxidant activities correlated highly. For the bars with autoclaved and fermented legumes, the antioxidant capacity was lower, except for the results of the ABTS assay. This parameter was higher by 10.9% for bars with fermented fava beans and 21.9% with autoclaved ones compared with SFB. According to the presented results, autoclaving reduced antioxidant activity in some legumes, but, on the contrary, in other cases they increased or were not affected [34]. These results depended on the types of legumes, processing parameters, and chosen assay and could also affect the obtained results for the final products.

### 3.3. Protein Characteristics of the Fava Bean-Based Bars

To develop a protein-rich product, much attention should be paid to protein quality characteristics due to their key contribution to the nutritional value of the final product.

The comparative analysis has shown that all bar samples were characterized by high content, followed by Asp, Arg, and Leu. This tendency aligns with the data presented by Muñoz-Pina et al. [19] and Mayer Labba et al. [52] for the fava beans’ amino acid profile. At the same time, the baking of bars, as one of the main technological processes, leads to a decrease of the amino acid concentrations for the final products compared with an initial amount of the plant-protein ingredient.

However, it was found that the processing of fava beans in different ways affected the amino acid composition of the bars. The results are summarized in Table 3. The bars based on the soaked fava beans compared with fermented ones were characterized by the higher level of Try and Trp—by 47.5% and 78.8%, respectively.

Autoclaved fava bean use increases some positively and negatively charged amino acids, such as Gly and Arg, compared with other samples. Adding the fermented fava beans to the snack recipe increases several essential amino acid concentrations compared with other samples. His concentration increased by 65.3%, Thr by 43.0%, and Ile by 31.2% compared with the soaked fava bean sample. Previously, Xie L. et al. [53] reported that a low level of His correlates with increased appetite and weight gain among children. Also, Kasaoka et al. [54] identified that its intake had suppressed retroperitoneal fat pad accumulation based on in vitro study results. Such a tendency is related to the metabolic conversion of the histidine to the histamine. It is known that it, as a neurotransmitter, plays an important role in food intake regulation [55]. The studies mentioned above showed that incorporating fermented fava beans as a main ingredient to the snack recipe could be an effective strategy for addressing the problem of gaining extra weight among children and adolescents. Lv X. et al. [56] have presented the results that not only branched-chain amino acids but also threonine and its metabolites may also have muscle protein synthesis-stimulating effects, which are vital for healthy child development.

The increase of the mentioned amino acids leads to a higher total amount of the essential amino acids by 8.6% for the FFB compared with the sample with soaked fava beans. Also, the essential amino acid (EAA) and non-essential amino acid (NEAA) ratios of SFB, AFB, and FFB were 0.5, 0.47, and 0.54%, respectively. It was also found that the samples with autoclaved and fermented fava beans were characterized by higher hydrophobic amino acid content compared with soaked ones. This group of amino acids has played a significant role in the binding and ability of the peptide to exhibit antibacterial activity and support the immune system in general [57].

The usage of the mixture of the developed ingredients (fava beans + puffed quinoa + pumpkin seeds) leads to the improvement of bars’ nutritional value in relation to their amino acid content compared with other presented studies. Thus, healthy snack bars supplemented with moringa sprout powder were characterized by lower EAA (28.01 ± 0.06 mg/g DM) and NEAA (49.97 ± 0.62 mg/g DM) content [58]. Tas et al. [36] also observed that the amount of the essential amino acids for the extruded protein-enriched snacks based on the mixture of corn, soybean, and chickpea flour was 59.18 mg/g of the product.

Also, considering the FAO/WHO requirements for protein and amino acid intake for young children’s nutrition [59], the usage of fermented fava beans in snack production, which leads to the increase of vital amino acid concentration, could be recommended as a beneficial product for children’s growth and development.

**Figure 2:**
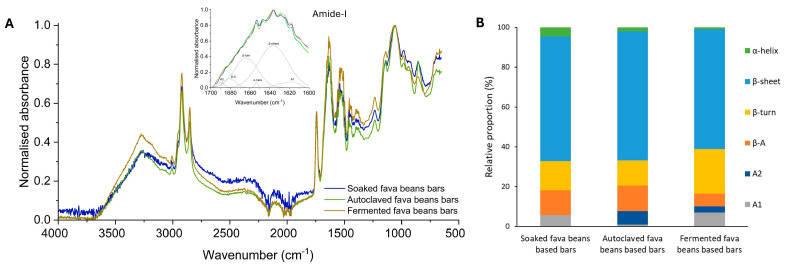
Fourier transform infrared spectroscopy analysis. (**A**) FTIR spectra of bar protein samples between 650 and 4000 cm^−1^. (**B**) Relative proportion of secondary structure of protein isolates from the fava bean-based bars. Green, α-helix; blue, β-sheet; yellow, β-turn; orange, anti-parallel β-sheet (β-A); dark blue, aggregates A2; grey, aggregates A1.

The Fourier transform infrared spectroscopy (FTIR) method was used to study the protein structure accompanying its conversion into an aggregated state. The obtained results allow for determining any modifications in bar protein conformation because of changes in fava bean processing methods. The obtained results could be closely associated with the functional properties of the main ingredients and, at the same time, influence the protein digestion process. The ATR-FTIR spectra of the bar samples are shown in Figure 2.

The five main absorption bands related to the main structure of proteins are identified in bar protein isolates, related to the amide A (3400 cm^−1^), amide B (3000 and 2800 cm^−1^), amide I (1630 cm^−1^), amide II (1530 cm^−1^), and amide III bands (1530 cm^−1^), respectively [60]. Therefore, the amide-I band was evaluated to determine the proportion of protein secondary structures for the fava bean-based bars. FTIR spectra analysis of protein form in the amide-I region revealed six major Gaussian bands centered at 1610–1625, ~1638, ~1654, ~1663, ~1680, and 1690–1695 cm^−1^, corresponding to A1, β-sheet, α-helix, β-turn, anti-parallel β-sheets (β-A), and A2 conformations, respectively [61]. The secondary structure of SFB isolates is mainly composed of β-sheet (62.8%), followed by β-turn (14.6%), β-A (12.5%), with a slight presence of aggregates (5.6%), and α-helix (4.5%). The results obtained are similar to those of Shevkani et al. [61], who pointed out the prevalence of the β-conformations rather than the α-helix structure for plant protein sources such as pulses.

The autoclaving process of fava beans leads to changes in their protein secondary structure. These samples were characterized with A2 presence (6.7%), which might be attributed to the protein aggregates and amino acid side-chains [62]. A decrease of the α-helix (2%) compared with bars based on the soaked fava beans was observed. The total amount of the protein β-structures (parallel and antiparallel β-sheets/β-turns) for this sample is 64.8, 12.9, and 12.7%, respectively. The reduction of the α-helix form for the bars with autoclaved fava beans could indicate that thermal treatment of the main ingredient increased protein aggregation of this form. Such a tendency could be due to the parameters of the autoclaving process. Huang et al. [63] have provided results revealing that long-term thermal treatment leads to lower protein α-helix structure formation in food matrixes. At the same time, it is revealed [64] that complex treatment with high temperature and high pressure is related to protein aggregation destruction with subsequent re-aggregation after processing. Also, based on the experimental results, the protein of AFB could be characterized as thermally more stable than those with a higher content of α-helix [62].

Regarding the protein secondary structure characteristics for the FFB, it was found that α-helix content was 0.9%, β-sheet was 60.3%, β-turn was 22.4%, and β-A was 6.3%. The rest was presented with aggregates A1 and A2. Adding fermented fava beans to the snack recipe decreases by 80% and 60% of the α-helix compared with SFB and AFB bars, which are characterized by a more uniform distribution of charges compared with β-sheet forms. Similar results were found by Muñoz-Pina et al. [19] for the fava beans fermented with the *Pleurotus ostreatus* strain and Zhao et al. [65] for the gluten powder fermented with the *Bacillus subtilis* strain. Such a tendency could be related to the action of fungus and fungal cross-linking enzymes, such as laccase, during the fermentation process. Its impact could result in the transformation of the main part of the stable α-helix structure to the irregular one due to the interruption of the hydrogen bonds between amino acid residues. At the same time, laccase, produced by *Pleurotus ostreatus*, promotes the formation of intramolecular bonds between biopolymers [66], affecting the protein secondary structure formation as well. The mentioned structure could affect the distribution of charges on the protein surface and, as a result, on the electrostatic interactions, and have great importance in maintaining the stability of the conformation of the protein [67]. Also, the FFB secondary protein structure with lower β-sheet content by 4…7% compared with other bars could be characterized by higher access to gastrointestinal digestive enzymes, as reported by Yu et al. [68], resulting in a higher protein value.

### 3.4. Impact of the Fava Beans Processing on the Protein Digestibility of Bars

The protein digestibility and content of amino acids of the bioaccessible fraction were studied to evaluate the effect of the fava bean processing method on the functional characteristics of the developed bars. The characteristic of protein digestibility for legume-based products is always an area of focus due to the known lower digestibility of macronutrients compared to animal proteins. Various researchers have reported the inherent difference in digestibility across legume sources related to the effect of processing methods [17,69]. The protein digestibility for the bar samples with soaked, autoclaved, and fermented fava beans was analyzed using the TCA-soluble protein test in the bioaccessible fraction after the in vitro gastrointestinal digestion. The obtained results were 39.4 ± 1.5 for SFB, 38.1 ± 1.8 for AFB, and 34.7 ± 2.4 g/100 g protein for FFB, respectively. It was found that the samples containing autoclaved and fermented fava beans were characterized by decreased protein hydrolysis by 3.3% and 12.0% compared with digested samples containing only soaked fava beans. The changes in AFB and FFB secondary protein structures, resulting in the formation of higher amounts of A1 and A2 aggregates, as shown in Figure 2, could affect the protein digestibility. This tendency was also observed by Muñoz-Pina et al. [19] when studying the effect of fermentation by *Pleurotus ostreatus* on the fava beans’ macronutrient digestibility.

Autoclaving fava beans, as one of the processing steps, is usually related to the Maillard reaction, which links the amino group of amino acids and the carbonyl group of reduced sugars. As a result of this reaction, the AFB and FFB were characterized with a more brownish color, according to the data in Table 3, which indicates the presence of melanoidins, products of the Maillard reaction, in their content. Despite the variety of beneficial effects of melanoidins, in vitro and in vivo studies have shown that melanoidins lower the activity of some proteolytic enzymes, including trypsin, affecting the degree of proteolysis during digestion [70].

Table 3 describes the profile of free amino acids resulting from proteolysis under the in vitro digestion model for the bar samples. Based on this, among the major amino acids for all digested samples were the presence of lysine and leucine, essential amino acids that are vital for children’s development according to WHO recommendations [59]. Also, it should be noted that all samples were characterized by a ratio EAA/NEEA value close to 1. Thus, the digested SFB was 0.96, AFB was 0.9, and FFB was 0.88, respectively—such results are related to the more favorable release of essential amino acids than non-essential ones. Previous work by our group showed the same tendency associated with the fava beans processed in several ways [19].

Regarding the effect of fava bean types of processing on the release of amino acids, digested AFB was characterized by no significant changes compared with the other samples. The digest from SFB had the higher content of Met (by 172%), Trp (by 79%), and Phe (by 33.7%). The digest of the FFB sample was characterized by higher (82.7% Glu, 58% Ser, 57.7% Val, 58.0% Ile, and 51%) Ala concentrations and increased NCAA in general. Based on the obtained results, it is noteworthy that Leu, Ile, and Val, as branched-chain amino acids, exerted beneficial health effects on obesity and its related metabolic dysfunction, according to the results presented by Huang et al. [71]. On the other hand, Siddik et al. [72] has given a review pointing out that, besides many physiological and metabolic benefits, branched-chain amino acids could be related to the pathophysiology of obesity and type 2 diabetes. Therefore, future recommendations for appropriate dietary branched-chain amino acids are required.

### 3.5. Effect of Fava Bean Treatment on the ACE Inhibition of the Bars (before and after Digestion)

Gaining extra weight for children is often accompanied by cardiometabolic dysfunction, such as an increase in hypertension. The development of cardiovascular diseases is correlated with the duration of obesity and could appear to pose more of a mortality risk than it does for adults [3,73]. Accordingly, enrichment for children and adolescents with food products characterized by a high content of angiotensin I-converting enzyme (ACE) inhibitors is necessary to prevent or reduce the risk of cardiovascular dysfunction development. Hypertension is one of the major independent risk factors for cardiovascular disease development and is indirectly caused by ACE. This enzyme catalyzes the conversion of peptide hormone to angiotensin II, which acts as a vasoconstrictor and contributes to increased blood pressure [32].

**Figure 3:**
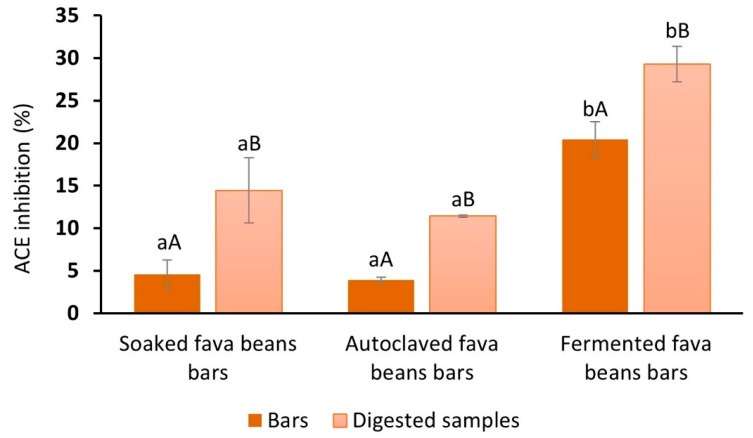
ACE inhibitory activity (%) of fava bean-based bars before and after GI digestion. a, b, Different lowercase letters indicate significant differences between bars (*p* < 0.05). A, B,: Different capital letters indicate significant differences between bars and digested samples (*p* < 0.05).

ACE inhibitory activity (%) was evaluated for bars with soaked, autoclaved, and fermented fava beans before and after gastrointestinal digestion (Figure 3). The addition of fermented *Pleurotus ostreatus* fava beans to the bar’s recipe provided a significant increase in their ACE inhibitory capacity. This indicator varied from 3.9% (with autoclaved fava beans) and 4.5% (with soaked fava beans) to 20.4% (with fermented fava beans). The study presented by Jakubczyk et al. [74] has shown that the fermentation of fava beans with *Lactobacillus plantarum* resulted in high-molecular-mass protein bands partially converted to peptide fractions with different sizes, affecting the inhibitory activity against ACE. This result is in close agreement with the findings of Sánchez-García et al. [73], with an observation about the greater ACE inhibitory capacities of lentils and quinoa fermented with *Pleurotus ostreatus*. Therefore, the solid-state fermentation of fava beans as a processing technology could influence the content and composition of the peptides, obtaining increased ACE inhibition capacity and positively affecting this parameter for the bar samples.

After in vitro digestion, the bioaccessible fraction of fermented fava bean bars also presented the highest level of ACE inhibition compared with other samples by approximately 15%. At the same time, ACE inhibition after digestion (29.3 ± 2.1%) was improved compared to the undigested sample with fermented legumes (20.4 ± 2.11%). The obtained data contradict the results presented by Diowksz et al. [75] regarding reducing ACE inhibition activity for sourdough bread from 93% without digestion to 59% after digestion. At the same time, our experimental result agreed with the study [76], which has shown that samples of whey and pea, fermented by *Lactobacillus helveticus* and *Saccharomyces cerevisiae*, in monoculture and combination, were characterized by a significant increase of the ACE inhibitory activity after the in vitro digestion process.

Based on this, it could be concluded that the ACE inhibitory activity of the food ingredients and final products is related to the type of processing for the used raw materials and the interaction between the fungus starter and suitable substrate in the case of fermentation. Using *Pleurotus ostreatus* for fava bean fermentation leads to the release of peptides, which exhibit potential physiological activity and significantly enhance ACE inhibition for the snacks before and after digestion.

### 3.6. Effect of Fava Beans Treatment on Some Mineral Bioaccessibility and Antioxidant Capacity of the Digested Samples

The 4–13-year-old childhood period is characterized by continued physical growth and rapid cognitive and emotional development. Due to increased growth and metabolism, the inadequate intake of micronutrients can negatively affect the growth and development of children. The nutritionally essential minerals, calcium, iron, and magnesium bioaccessibility for the bar samples were studied, and the results are presented in Table 4. It was found that the digestion process did not provide a total release of Mg and Ca. At the same time, the highest level of Mg bioaccessibility was found in the digest from FFB (24%) compared with AFB (15%) and SFB (16%) digested samples. Because increasing the consumption of high dietary magnesium foods might favor children’s body mass index [77], enrichment snacks with fermented fava beans by *Pleurotus ostreatus* look effective for children’s weight management. The reverse tendency was presented for Ca bioaccessibility, in which low intake during childhood could be related to a greater risk for increased body fat and risk for osteoporosis later in life [78]. In this case, the autoclaving and fermenting of fava beans significantly decrease the release of the Ca in bars digested by 14% compared with SFB. The same tendency was observed by Muñoz-Pina et al. [19] for the impact of fava bean treatment, revealing antagonistic properties of Mg to Ca. The most positive impact on mineral bioaccessibility was observed in terms of Fe. The level of its bioaccessibility was 100, 83, and 79% for SFB, AFB, and FFB, respectively. Based on the obtained results, fava beans seem to be a prospective ingredient for fortifying snacks with Mg, Ca, and Fe.

A significant increase in the TPC bioaccessibility was also observed for all digested samples (Table 4). Compared to the undigested samples of bars, this parameter of the digested samples was higher by 3 to 4 times depending on the type of fava beans used. The highest content of the liberated phenolics was for FFB. This could be related to the role of fermentation during fava bean processing, resulting in higher hydrolytic enzyme activity, which could lead to the increased bioaccessibility of phenolics for the bars.

Despite the higher TPC content, the digest of FFB was characterized by lower antioxidant capacity by DPPH and FRAP assays. The loss of antioxidant activity may be due to synergistic combinations or interactions of different types of chemical reactions, the diffusion of water-soluble compounds, and their formation or degradation, as mentioned by Mencin et al. [79], observing the same tendency for the spelled-enhanced wheat bread after digestion.

## 4. Conclusions

This study evaluated the effect of soaking, autoclaving, and fermentation by *Pleurotus ostreatus* on the bars’ quality and nutritional characteristics. It can be concluded that the fava bean could be a potential ingredient in producing protein-rich bars. Various fava bean processing methods lead to changes in the bars’ characteristics. Thus, adding autoclaved fava beans to the bar recipe improves its mineral content, especially Ca and Mg. According to the preliminary evaluation, samples based on autoclaved and fermented legumes performed better sensory attributes, such as color, aroma, and taste, compared with bars based on soaked fava beans. In the future, it will be necessary to examine in detail the impact of the fava bean processing methods on the organoleptic characteristics of the bars, involving trained panelists and target consumers (children and their parents).

Additionally, the finding revealed that fermented fava beans provide a higher content of essential amino acids, especially His, Thr, and Ile, to bars, reducing the presence of antinutrient compounds. Also, undigested and digested samples containing fermented fava beans were characterized by higher angiotensin-converting enzyme inhibition activity and improved preliminary sensorial characteristics compared with other samples. All digested samples were characterized with high Fe bioaccessibility and significantly improved TPC bioaccessibility compared with undigested samples.

Based on the obtained results, it was demonstrated that adding fava beans as a healthy and nutritious ingredient could be promising for snack production. In this study, we discovered, for the first time, the impact of the fermented fava beans by *Pleurotus ostreatus* on the snack’s functional and qualitative characteristics and revealed its potential as the main component for plant protein product design to prevent children from becoming overweight and improve the general health of other usual consumers of these types of foodstuffs. However, the ratio of the ingredients requires future investigation and adjustments to enhance the developed snacks’ antioxidant activity and protein digestibility.

## Figures and Tables

**Table 1:** Color coordinates and hardness of fava bean-based bars.

	Soaked Fava Bean-Based Bars	Autoclaved Fava Bean-Based Bars	Fermented Fava Bean-Based Bars
Color			
L*	42.8 ± 1.5 ^c^	34.8 ± 1.3 ^a^	37.3 ± 1.5 ^b^
a*	12.1 ± 0.3 ^b^	11.3 ± 0.7 ^ab^	10.7 ± 0.3 ^a^
b*	28.3 ± 1.3 ^b^	23.13 ± 0.30 ^a^	24.5 ± 1.0 ^a^
C*	30.8 ± 1.3 ^b^	25.7 ± 0.8 ^a^	27.300 ± 0.009 ^a^
h	66.7 ± 0.4 ^b^	64.0 ± 0.9 ^a^	67.0 ± 0.8 ^b^
∆E	-	9.6 ± 0.7 ^b^	6.2 ± 1.5 ^a^
Hardness (g)	731 ± 54 ^b^	691 ± 29 ^ab^	572 ± 16 ^a^

Results represent the mean of three repetitions with their standard deviation. Different lowercase letters indicate significant differences with a 95% confidence level (*p* < 0.05). L*, lightness; +a*, redness; −a*, greenness; +b*, yellowness; −b*, blueness; h, hue angle; C*, chroma; ∆E, total color difference.

**Table 2:** Proximate composition, calcium, iron, magnesium, tannins, total phenolic compounds, and antioxidant capacity (DPPH, ABTS, FRAP) of fava bean-based bars.

Nutrient	Soaked Fava Bean-Based Bars	Autoclaved Fava Bean-Based Bars	Fermented Fava Bean-Based Bars
Major components			
Moisture (g/100 g)	28.3 ± 1.4 ^b^	24.6 ± 0.5 ^a^	23.8 ± 0.5 ^a^
Protein (g/100 g DB)	23.8 ± 0.7 ^a^	22.1 ± 1.5 ^a^	25.1 ± 0.3 ^a^
Lipid (g/100 g DB)	12.2 ± 0.7 ^a^	12.1 ± 0.5 ^a^	12 ± 2 ^a^
Carbohydrates (g/100 g DB)	61 ± 3 ^a^	63.2 ± 4 ^a^	60 ± 4 ^a^
Reducing sugar	4.3 ± 0.2 ^b^	3.9 ± 0.8 ^a^	4.9 ± 0.3 ^c^
Total starch	33.7 ± 0.3 ^a^	39.2 ± 1.1 ^a^	38.5 ± 1.5 ^a^
Ashes (g/100 g DB)	2.8 ± 0.1 ^ab^	2.7 ± 0.2 ^a^	3.1 ± 0.1 ^b^
Minor components			
Calcium (mg/100 g)	122 ± 6 ^a^	147 ± 30 ^a^	115 ± 7 ^a^
Iron (mg/100 g)	3.6 ± 0.2 ^a^	2.98 ± 0.01 ^a^	3.8 ± 0.7 ^a^
Magnesium (mg/100 g)	230 ± 4 ^a^	285 ± 10 ^a^	250 ± 20 ^a^
Total phenols content (mg GAE/g DB)	3.71 ± 0.18 ^b^	2.99 ± 0.15 ^a^	2.4 ± 0.3 ^a^
Antioxidant capacity (mg TE/g DB)			
- DPPH	5.7 ± 0.8 ^c^	4.8 ± 0.7 ^b^	4.0 ± 0.3 ^a^
- ABTS	1.0 ± 0.5 ^a^	1.28 ± 0.06 ^c^	1.14 ± 0.09 ^b^
- FRAP	10.0 ± 0.6 ^b^	7.4 ± 0.2 ^a^	6.3 ± 0.3 ^a^
Tannins (mg CAE/g DB)	0.97 ± 0.01 ^c^	0.85 ± 0.02 ^b^	0.79 ± 0.02 ^a^
Energy value (kcal/100 g)	357.0	364.5	368.0

Results represent the mean of three repetitions with their standard deviation. ^a^, ^b^, ^c^: Different lowercase letters indicate significant differences between bar samples with a 95% confidence level (*p* < 0.05).

**Table 3:** Amino acid profile (mg free AA/g dry basis) of undigested and digested fava bean-based bars.

Amino Acid (mg Free AA/g DB)	Undigested			Digested		
	Soaked Fava Bean-Based Bars	Autoclaved Fava Bean-Based Bars	Fermented Fava Bean-Based Bars	Soaked Fava Bean-Based Bars	Autoclaved Fava Bean-Based Bars	Fermented Fava Bean-Based Bars
Aspartic acid (Asp)	18 ± 1.0 ^a^	20.7 ± 0.2 ^a^	19.9 ± 1.3 ^a^	1.43 ± 0.2 ^a^	1.0 ± 0.4 ^a^	2.0 ± 0.2 ^a^
Serine (Ser)	8.3 ± 0.2 ^a^	9.2 ± 0.5 ^a^	8.1 ± 0.5 ^a^	3.4 ± 0.5 ^a^	3.4 ± 0.5 ^a^	5.3 ± 0.6 ^b^
Glutamic acid (Glu)	29 ± 2 ^a^	36.0 ± 0.6 ^b^	30.3 ± 0.5 ^a^	3.4 ± 0.4 ^a^	3.4 ± 0.3 ^a^	6.3 ± 0.6 ^b^
Glycine (Gly)	10.4 ± 0.8 ^a^	12.7 ± 0.3 ^a^	14 ± 1.0 ^a^	2.1 ± 0.3 ^a^	2 ± 1.0 ^a^	3.1 ± 0.3 ^b^
Histidine (His)	3.7 ± 0.3 ^a^	4.67 ± 0.08 ^b^	6.13 ± 0.10 ^c^	3.8 ± 0.3 ^a^	3.02 ± 1.2 ^a^	4.2 ± 0.5 ^a^
Arginine (Arg)	21 ± 1.0 ^b^	21.9 ± 0.4 ^b^	15.7 ± 1.3 ^a^	10.0 ± 0.5 ^a^	7.1 ± 1.4 ^a^	8.0 ± 1.0 ^a^
Threonine (Thr)	4.2 ± 0.3 ^a^	4.0 ± 0.3 ^a^	6.0 ± 0.4 ^b^	1.0 ± 0.3 ^a^	1.3 ± 0.2 ^a^	1.9 ± 0.5 ^a^
Alanine (Ala)	7.4 ± 0.5 ^a^	8.66 ± 0.02 ^b^	9.0 ± 0.4 ^b^	2.1 ± 0.2 ^a^	2.1 ± 0.9 ^a^	3.1 ± 0.3 ^b^
Proline (Pro)	7.8 ± 0.5 ^a^	7.2 ± 0.2 ^a^	7.7 ± 0.4 ^a^	1.2 ± 0.1 ^a^	1.4 ± 0.6 ^ab^	1.8 ± 0.1 ^b^
Cystine (Cys)	1.66 ± 0.02 ^a^	1.61 ± 0.05 ^a^	1.61 ± 0.06 ^a^	0.32 ± 0.02 ^a^	0.4 ± 0.1 ^a^	0.52 ± 0.06 ^a^
Tyrosine (Try)	4.5 ± 0.3 ^b^	3.50 ± 0.06 ^a^	3.1 ± 0.3 ^a^	4.2 ± 0.4 ^a^	3.3 ± 0.4 ^a^	4.0 ± 0.5 ^a^
Valine (Val)	7.7 ± 0.7 ^a^	8.6 ± 0.1 ^a^	8.1 ± 0.3 ^a^	2.6 ± 0.2 ^a^	2.6 ± 1.4 ^a^	4.1 ± 0.5 ^b^
Methionine (Met)	1.73 ± 0.03 ^a^	1.4 ± 0.2 ^a^	1.1 ± 0.2 ^a^	0.60 ± 0.06 ^b^	0.22 ± 0.09 ^a^	0.42 ± 0.08 ^ab^
Lysine (Lys)	9.4 ± 0.7 ^a^	10.2 ± 0.3 ^a^	8.6 ± 0.6 ^a^	6.3 ± 0.6 ^a^	4.2 ± 1.6 ^a^	5.8 ± 0.7 ^a^
Isoleucine (Ile)	5.6 ± 0.4 ^a^	6.54 ± 0.04 ^ab^	7.3 ± 0.2 ^b^	2.1 ± 0.2 ^a^	2.2 ± 1.1 ^a^	3.3 ± 0.3 ^b^
Leucine (Leu)	11.9 ± 0.8 ^a^	12.75 ± 0.08 ^a^	12.0 ± 1.0 ^a^	5.9 ± 0.5 ^a^	5.0 ±1.0 ^a^	6.2 ± 0.7 ^a^
Phenylalanine (Phe)	7 ± 1.0 ^a^	8.2 ± 0.2 ^a^	8.5 ± 0.4 ^a^	3.7 ± 0.3 ^b^	2.7 ± 1.1 ^a^	3.5 ± 0.4 ^b^
Tryptophan (Trp)	2.5 ± 0.2 ^c^	1.8 ± 0.1 ^b^	1.4 ± 0.2 ^a^	1.1 ± 0.2 ^b^	0.9 ± 0.4 ^ab^	0.62 ± 0.07 ^a^
EAA	54 ± 4 ^a^	57 ± 2 ^a^	58 ± 3 ^a^	27 ± 3 ^a^	22 ± 8 ^a^	30 ± 4 ^a^
NEAA	108 ± 7 ^a^	122 ± 3 ^b^	109 ± 6 ^a^	28 ± 3 ^a^	24 ± 4 ^a^	34 ± 4 ^a^
EAA/NEAA ratio	0.50 ± 0.05 ^a^	0.47 ± 0.04 ^a^	0.54 ± 0.05 ^a^	0.96 ± 0.05 ^a^	0.9 ± 0.1 ^a^	0.88 ± 0.06 ^a^
HAA	58 ± 5 ^a^	59.3 ± 1.4 ^a^	59 ± 4 ^a^	23.9 ± 1.7 ^a^	21 ± 7 ^a^	28 ± 3 ^a^
PCAA	33.4 ± 2 ^a^	36.7 ± 0.8 ^b^	30 ± 2 ^a^	20.1 ± 1.4 ^a^	14 ± 4 ^a^	18 ± 2 ^a^
NCAA	47.4 ± 1.8 ^a^	56.7 ± 0.5 ^c^	50 ± 2 ^b^	4.9 ± 0.6 ^a^	4.5 ± 0.7 ^b^	8.2 ± 0.8 ^b^
AAA	14.1 ± 1.5 ^b^	12.5 ± 0.4 ^a^	12.3 ± 0.9 ^a^	9.0 ± 0.9 ^b^	6.9 ± 1.9 ^a^	8.2 ± 1.0 ^b^

The results represent the mean of three repetitions. ^a^, ^b^, ^c^: Different lowercase letters indicate significant differences between bars (*p* < 0.05). Essential amino acids (EAA); Nonessential amino acids ratio (NEAA); Hydrophobic amino acids (HAA) = Ala, Pro, Cys, Tyr, Val, Met, Ile, Leu, Phe, and Trp; Positively charged amino acids (PCAA) = His, Arg, and Lys; Negatively charged amino acids (NCAA) = Asp and Glu; Aromatic amino acids (AAA) = Tyr, Phe, and Trp.

**Table 4:** Mineral content, total phenolic content, and antioxidant capacity (by DPPH, FRAP, and ABTS tests) in the bioaccessible fraction of fava bean-based bar digesta under standard in vitro GI conditions.

	Mineral Release (mg/100 g DB)			Total Phenolic Content (mg GAE/g DB)	Antioxidant Capacity (mg TE/g DB)			
	Mg	Ca	Fe		DPPH	FRAP	ABTS	APCI *
UFB	37 ± 4 ^a^ (16)	25 ± 7 ^b^ (20)	4.0 ± 0.6 ^b^ (100)	11.7 ± 0.2 ^b^ (315)	10.7 ± 0.4 ^b^ (90)	2.0 ± 0.2 ^c^ (100)	3.90 ± 0.07 ^c^ (100)	96.7
AFB	41 ± 9 ^a^ (15)	11.8 ± 1.9 ^ab^ (6)	2.4 ± 0.8 ^a^ (83)	10.4 ± 0.7 ^a^ (348)	11.9 ± 0.6 ^c^ (100)	1.40 ± 0.02 ^b^ (70)	3.5 ± 0.1 ^a^ (90)	86.7
FFB	77 ± 8 ^b^ (24)	14.2 ± 0.6 ^a^ (6)	3.0 ± 0.3 ^ab^ (79)	10.1 ± 0.5 ^a^ (414)	2.3 ± 0.01 ^a^ (19)	0.8 ± 0.1 ^a^ (40)	3.75 ± 0.02 ^b^ (96)	51.7

The data shown are mean values from three replicates. Values in parentheses in mineral release and total phenolic content represent the bioaccessibility (%). Values in parentheses in antioxidant capacity represent the antioxidant index (%). a, b, c: Lowercase letters indicate significant differences between the fava beans at a significance level of 95% (*p* < 0.05). * APCI: Antioxidant potency composite index. UFB—soaked fava bean-based bars; AFB—autoclaved fava bean-based bars; FFB—fermented fava bean-based bars.

## Data Availability

All related data and methods are presented in this paper. Additional inquiries should be addressed to the corresponding author.
